# Effect of age on pharmacokinetics, efficacy, and safety of galcanezumab treatment in adult patients with migraine: results from six phase 2 and phase 3 randomized clinical trials

**DOI:** 10.1186/s10194-020-01148-9

**Published:** 2020-06-23

**Authors:** Virginia L. Stauffer, Ira Turner, Phebe Kemmer, William Kielbasa, Kathleen Day, Martha Port, Tonya Quinlan, Angelo Camporeale

**Affiliations:** 1 Lilly Research Laboratories, Lilly Corporate Center, Indianapolis, IN USA; 2 Center for Headache Care and Research, Island Neurological Associates, a division of ProHEALTHcare Associates, Plainview, NY USA; 3 Eli Lilly Italia, Sesto Fiorentino, Italy

**Keywords:** Aging population, Elderly, Migraine, CGRP antagonist, Migraine in older adults, Migraine prevention, Migraine prophylaxis, Galcanezumab, Monoclonal antibody

## Abstract

**Background:**

Migraine clinical profile may change with age, making it necessary to verify that migraine treatments are equally safe and effective in older patients. These analyses evaluated the effects of patient age on the pharmacokinetics (PK), efficacy, and safety of galcanezumab for prevention of migraine.

**Methods:**

Analyses included efficacy data from three double-blind phase 3 clinical trials: two 6-month studies in episodic migraine (EVOLVE-1, EVOLVE-2: *N* = 1773) and one 3-month study in chronic migraine (REGAIN:*N* = 1113). Patients were randomized 2:1:1 to placebo, galcanezumab 120 mg, or galcanezumab 240 mg. Safety and PK data included additional phase 2 and phase 3 trials for a larger sample size of patients > 60 years (range = 18–65 for all studies). Subgroup analyses assessed efficacy measures, adverse event (AE) occurrence, and cardiovascular measurement changes by patient age group. Galcanezumab PK were evaluated using a population analysis approach, where age was examined as a potential covariate on apparent clearance (CL/F) and apparent volume of distribution (V/F) of galcanezumab.

**Results:**

Numbers of baseline monthly migraine headache days were similar across age groups. There were no statistically significant treatment-by-age group interactions for any efficacy measures, except in episodic migraine studies where older patients appeared to have a larger reduction than younger patients in the number of monthly migraine headache days with acute medication use. Age (18–65) had a minimal effect on CL/F, and no effect on V/F. Galcanezumab-treated patients ≥60 years experienced no clinically meaningful increases in blood pressure and no increased frequency in treatment-emergent AEs, discontinuations due to AEs, serious adverse events (SAEs) overall, or cardiovascular SAEs, compared to age-matched placebo-treated patients.

**Conclusions:**

Age (up to 65 years) does not affect efficacy in migraine prevention and has no clinically meaningful influence on galcanezumab PK to warrant dose adjustment. Furthermore, older galcanezumab-treated patients experienced no increases in frequency of AEs or increases in blood pressure compared with age-matched placebo-treated patients.

**Trial registrations:**

EVOLVE-1 (NCT02614183, registered 23 November 2015), EVOLVE-2 (NCT02614196, 23 November 2015), REGAIN (NCT02614261, 23 November 2015), ART-01 (NCT01625988, 20 June 2012, ), I5Q-MC-CGAB (NCT02163993, 12 June 2014, ), I5Q-MC-CGAJ (NCT02614287, 23 November 2015, ), all retrospectively registered.

## Background

Galcanezumab is a humanized monoclonal antibody (mAb) that targets calcitonin gene-related peptide (CGRP) and is currently approved for migraine prevention in many countries including the United States and those of the European Union [1, 2]. Galcanezumab is injected subcutaneously once per month and reduces the number of monthly migraine headache days in patients with both episodic and chronic migraine [1–5].

While migraine is most prevalent in people aged 25 to 55 years [6], many people over the age of 55 years also experience migraine attacks [7–9], with the one-year prevalence of migraine among older adults being reported as approximately 10% [10]. However, diagnosis of migraine typically occurs before the age of 50 years and the prevalence of migraine tends to decrease with age [10, 11].

Population pharmacokinetic (PK) analyses are commonly used to understand the influence of age on drug exposure, efficacy, and safety to help guide clinical dosing regimens. A population PK analysis examining the effect of age on apparent clearance (CL/F) and apparent volume of distribution (V/F) of galcanezumab can provide useful data for health professionals in understanding the PK risk of the drug in patients of various ages.

Because aging is associated with cardiovascular changes, resulting in altered blood pressure (BP) and heart rate [12], potential cardiovascular effects of a drug are of increased concern when considering treatment options for an older population. Some migraine medications, such as ergots and triptans, are contraindicated in patients with certain cardiovascular and cerebrovascular conditions, due to their vasoconstrictive effects [13]. As galcanezumab blocks the action of CGRP, which is a potent microvascular vasodilator with a number of physiological roles [14], it is necessary to determine if galcanezumab treatment increases occurrences of cardiovascular-related adverse events or high BP, specifically in older patients.

Furthermore, how migraine is expressed can change with age, and these changes could affect the way a patient responds to medication. A trend towards less severe migraine attack has been reported in patients over 50 years old [15], and migraine profile can change as a person gets older, with some people reporting improvement, some worsening, and some developing a less typical migraine profile [15, 16].

The purpose of this analysis was to determine the effects of age on PK, efficacy, and safety of galcanezumab treatment in patients with episodic and chronic migraine.

## Methods

### Clinical trials

The analyses by age used data from six clinical trials, including three phase 3 placebo-controlled, double-blinded trials, two phase 2 placebo-controlled, double-blinded trials, and one open-label phase 3 trial. All trials mentioned in this analysis complied with the Declaration of Helsinki and followed the guidelines of the International Conference on Harmonization on Good Clinical Practices and all applicable laws and regulations. Studies were approved by each institution’s ethical review board and all trial participants provided written, informed consent prior to enrollment. All trials enrolled patients ≥18 to ≤65 years, who had been diagnosed with migraine for at least 1 year (for episodic migraine only) and prior to the age of 50 years.

Detailed descriptions for the study designs for EVOLVE-1, EVOLVE-2, and REGAIN have been described previously [3–5], as have the additional clinical trials included in the PK and safety populations [17–19]. Key points of the six trials are summarized in Tables 1.

**Table 1:** Clinical trials included in analyses

**Phase 3 placebo-controlled trials**			
	**EVOLVE-1**	**EVOLVE-2**	**REGAIN**
NCT number	NCT02614183	NCT02614196	NCT02614261
No. of patients randomized and treated (ITT)	858	915	1113
Study centers	90 in US and Canada	109 in Asia, Europe, North and South America	116 in Asia, Europe, North and South America
Headache frequency	4-14 migraine headache days/month (episodic migraine)		≥15 headache days/month^a^ (chronic migraine)
Double-blind	6 months		3 months
Open label	None		9 months
Additional migraine preventive medications	Not permitted		Stable doses of allowed treatments permitted^b^
Trial phase	3		
PBO-controlled	Yes		
Treatment arms	GMB 120 mg with 240 mg loading dose GMB 240 mg PBO		
Treatment regimen	Subcutaneous injection every month		
**Other clinical trials**			
	**ART-01**	**CGAB**	**CGAJ**
NCT number	NCT01625988	NCT02163993	NCT02614287
No. of patients randomized and treated (ITT)	217	410	270
Study centers	35 in US	37 in US	28 in North America and Europe
Headache frequency	4 – 14 migraine headache days/28 days	4 – 14 migraine headache days/28 days	≥4 migraine headache days/month
Double-blind	12 weeks	12 weeks	none
Open label	None	None	12 months
Additional migraine preventive medications	Not permitted	Not permitted	Not permitted
Trial phase	2	2b	3
PBO-controlled	Yes	Yes	No
Treatment arms	GMB 150 mg PBO	GMB 300 mg GMB 120 mg GMB 50 mg GMB 5 mg PBO	GMB 120 mg GMB 240 mg
Treatment regimen	Subcutaneous injection every 2 weeks	Subcutaneous injection every 28 days	Subcutaneous injection every month

*Abbreviations*: *GMB* galcanezumab, *ITT* intent-to-treat, *NCT* national clinical trial, *PBO* placebo

^a^At least eight of the monthly headache days were migraine headache days

^b^Permitted migraine preventive medications included topiramate and propranolol

### Analyses by age

Different patient populations and clinical trials were used for the age analyses described in this manuscript (Table 2). Data from the three phase 3 placebo-controlled trials (EVOLVE-1, EVOLVE-2, and REGAIN) were used for baseline comparisons and analyses of efficacy outcomes. Pharmacokinetic analyses included patients from EVOLVE-1, EVOLVE-2, REGAIN, the open-label extension of REGAIN, a phase 2 placebo-controlled trial (CGAB), and a phase 3 open-label trial (CGAJ). Safety outcomes were analyzed using two populations: 1) *Phase 3_Pooled* population, which included both galcanezumab- and placebo-treated patients, and 2) *All GMB Exposure* population, which included only galcanezumab-treated patients from all trials.

**Table 2:** Clinical trial populations included in analyses

Analysis	Clinical Trials					
	EVOLVE-1 Phase 3 DB	EVOLVE-2 Phase 3 DB	REGAIN Phase 3 DB and OL	ART-01 Phase 2 DB	CGAB Phase 2 DB	CGAJ Phase 3 OL
Pharmacokinetic^a^	X	X	X		X	X
Baseline comparison	X	X	X			
Efficacy outcomes	X	X	X^b^			
Safety outcomes						
Phase 3_Pooled	X	X	X^b^			
All GMB Exposure	X	X	X	X	X	X

*Abbreviations*: *All GMB Exposure* Patients treated with any GMB dose in any duration, *DB* Double blind, *GMB* Galcanezumab, *OL* Open label, Phase 3_Pooled, all patients from the 3 placebo-controlled phase 3 trials

^a^Includes results from phase 2 and 3 studies with 28-day or monthly dosing regimens

^b^Includes results from DB phase of trials only

### Pharmacokinetic analyses

A prior population PK analysis using PK data obtained from healthy adults and adult patients with migraine dosed with 5 to 300 mg galcanezumab showed that the typical population estimate of CL/F was 0.00785 L/h with 34% inter-individual variability (IIV), and the typical population estimate of V/F was 7.33 L with 34% IIV [20]. In the current study, age was examined as a potential continuous covariate on CL/F and V/F of galcanezumab using a covariate power model (Eq. 1), exponential model (Eq. 2), and linear model (Eq. 3) as shown below:

1 $$ \mathrm{P}=\Theta 1\times {\left(\mathrm{COV}/\mathrm{MED}\right)}^{\Theta 2} $$

2 $$ \mathrm{P}=\Theta 1\times {\mathrm{e}}^{\Theta 2\left[\mathrm{COV}-\mathrm{MED}\right]} $$

3 $$ \mathrm{P}=\Theta 1\ \left(1+\Theta 2\ \left[\mathrm{COV}-\mathrm{MED}\right]\right) $$

where P is the estimate of CL/F or V/F for an individual patient, Θ1 represents the typical value of CL/F or V/F in the patient population, Θ2 represents the effect of age, COV is the patient age, and MED is the population median age. Details of the covariate analyses can be found elsewhere [20]. Briefly, the effect of age was considered to be statistically significant if the value of the model objective function (MOF) after testing age as a covariate was reduced by 6.635 points (*p*-value < 0.01) and there was a > 5% decrease in the IIV of CL/F or V/F compared to the typical base model IIV values of 40% for CL/F and 34% for V/F.

### Analyses of baseline characteristics and efficacy outcomes

#### Clinical trials and age groupings

Efficacy outcomes and corresponding baseline frequencies were analyzed for the patient populations in the double-blind sections of the three phase 3, placebo-controlled trials. Patients from EVOLVE-1 and EVOLVE-2 trials were pooled to make a single population of patients with episodic migraine and were analyzed separately from the population of patients with chronic migraine (REGAIN). Both patient groups were divided into 4 age groups, ≤40, > 40 to ≤50, > 50 to ≤55, and > 55 to ≤65 years. Patients older than 50 years were divided into age groups of > 50 to ≤55 years and > 55 to ≤65 years in order to have the two groups include comparable numbers of patients.

#### Baseline characteristics

Baseline levels for monthly migraine headache days and monthly migraine headache days with acute medication use were determined during the prospective baseline period lasting 30–40 days before the start of the double-blind treatment phase. Baseline health outcome measures including Migraine-Specific Quality-of-life Questionnaire v2.1 (MSQ), and Migraine Disability Assessment (MIDAS) scores were also collected to cover the baseline period. The MSQ measures a person’s level of functioning, with a higher score indicating better function. The MIDAS assesses the level of headache-related disability due to migraine, with a higher score indicating more disability [21]. Trial participants also reported patient global impression of severity (PGI-S), a measure that had the patient answer the question “Considering migraine as a chronic condition, how would you rate your level of illness?”, on a scale of 1 (“normal, not at all ill”) to 7 (“extremely ill”).

#### Statistical analyses for baseline characteristics

For all baseline patient and migraine characteristics, the overall means of each age group were compared using an analysis of variance model, with terms for age group and study for the episodic trials and only age group for the chronic trial.

#### Efficacy outcomes

Efficacy outcomes evaluated in this analysis by age were the overall mean changes from baseline in monthly migraine headache days and monthly migraine headache days with acute medication use. Percentage of patients experiencing ≥50% and ≥ 75% reductions from baseline in monthly migraine headache days were also calculated using the average over all months in the trials.

#### Statistical analyses for efficacy outcomes

The overall mean change from baseline in the number of migraine headache days and migraine headache days with acute medication use per month by age group were analyzed using mixed model for repeated measures approach both for age subgroup-by-treatment interaction and treatment comparisons within each age group. To analyze ≥50% and ≥ 75% response rates by age group, generalized linear mixed model was used both for age subgroup-by-treatment interaction and treatment comparisons within each age group.

### Analyses for safety outcomes

#### Safety datasets and age groupings

Safety outcomes were examined for two sets of patients, the “double-blind treatment phase” dataset (Phase 3_Pooled) and the “all galcanezumab exposure” dataset (All GMB Exposure), defined in Table 2. Patients were divided into 4 age groups using the median age of 42 years as the upper limit for the youngest age group, resulting in the patient subgroups of < 42, 42 to < 50, 50 to < 60, and 60 to ≤65 years of age. These groups were slightly different than those used in the efficacy analyses, with the most important difference being in the age range for the oldest group (60 to ≤65 years instead of > 55 to ≤65 years), which allowed for a more specific analysis of the safety of galcanezumab in the oldest patients in the trials.

#### Safety outcomes

Safety outcomes evaluated in this subgroup analysis included number of treatment-emergent adverse events (TEAEs), serious adverse events (SAEs), and discontinuations due to adverse events (DCAEs). TEAEs were defined as adverse events that occurred for the first time or worsened in severity after the baseline period. Specific TEAEs that were likely to be cardiovascular in nature were evaluated using the standardized Medical Dictionary for Regulatory Activities (MedDRA version 19.1) queries (SMQs), including only narrow terms, for hypertension, embolic and thrombotic events, and ischemic heart disease. Occurrences of high systolic and diastolic BP were determined for each age group. An increased (high) BP postbaseline was defined as ≥140 mmHg for systolic BP and ≥ 90 mmHg for diastolic BP measured at any time after the baseline period, with a concomitant increase of ≥20 mmHg (systolic BP) and ≥ 10 mmHg (diastolic BP), from the baseline BP.

#### Statistical analyses performed for safety outcomes

Exposure-adjusted incidence rates (EAIRs) and unadjusted percentages are presented for all safety outcomes for both the “Phase 3_Pooled” and “All GMB Exposure” populations. An EAIR is calculated as 100 times the number of patients experiencing the specific event, divided by the event-specific total patient-year-at-risk. Patient-year-at-risk accrues up to the date of the first occurrence of the event for patients with the event, and to the end of the specified study period for patients without the event.

For the Phase 3_Pooled population, exposure-adjusted analyses of safety outcomes within an age subgroup used a Poisson regression model with explanatory variables of study and treatment and an offset term of time-at-risk. *P*-values comparing galcanezumab and placebo treatment groups were determined using a likelihood ratio test of the treatment effect. Treatment-by-age group interaction for safety outcomes was evaluated using a Poisson regression model with explanatory variables of study, treatment, age group, and treatment-by-age group interaction, with the offset term of time-at-risk.

For the All GMB Exposure population, only galcanezumab-treated patients were combined for analysis; no *p*-values are presented since there were no treatment comparisons.

## Results

### Pharmacokinetics

**Fig. 1:**
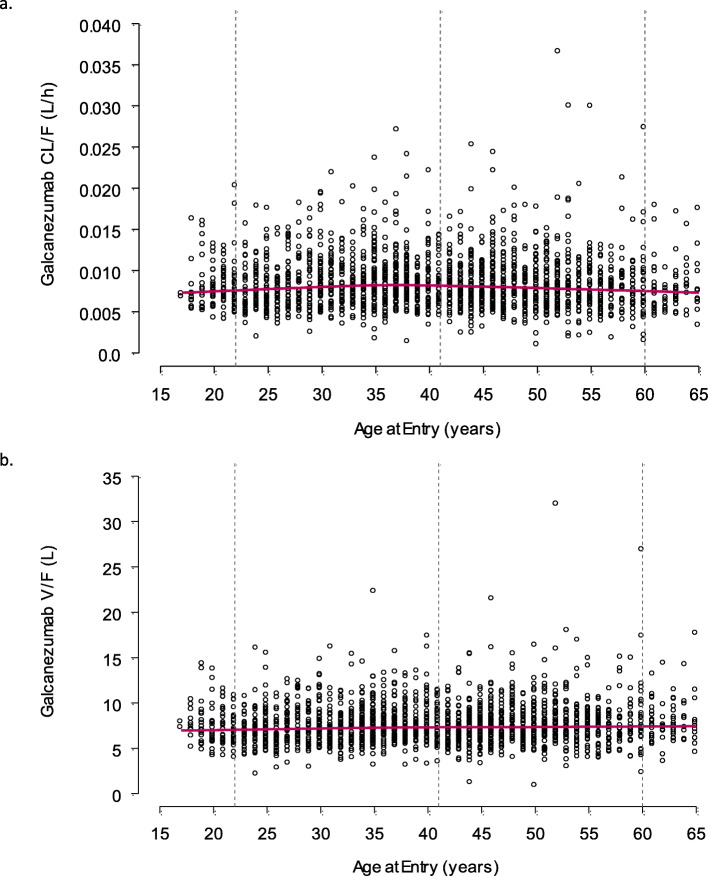
Effect of age (18–65 years) on galcanezumab CL/F and V/F

Covariate analysis revealed that age had a statistically significant effect on CL/F using the power model (*p* < 0.01, reduction in MOF of 13) and the exponential model (*p* < 0.01, reduction in MOF of 20), but not the linear model (*p* > 0.01, increase in MOF of 21). However, the IIV for CL/F was unchanged from 40% when age was included in the population PK model. Since the pre-specified criteria for age as a covariate on galcanezumab CL/F was a decrease of 6.635 points (*p*-value < 0.01) in the MOF and a decrease in the IIV of the parameter by > 5%, the criteria overall were not met. The relationship between age and CL/F is illustrated in Fig. 1a, demonstrating a minimal change across patients who were between 18 and 65 years of age. The CL/F was 0.00842 L/h at 18 years and 0.00739 L/h at 65 years based on the exponential model, resulting in a minimal decrease of 14% in CL/F from the youngest to the oldest patients evaluated. Overall, age was not considered a clinically meaningful covariate on galcanezumab CL/F.

For V/F, age did not have a statistically significant effect using the power model (*p* > 0.01, reduction in MOF of 18), exponential model (*p* > 0.01, increase in MOF of 11), or linear model (*p* > 0.01, reduction in MOF of 1.4). Therefore, age was not considered to be a meaningful covariate on galcanezumab V/F. For completeness, the relationship between age and V/F is illustrated in Fig. 1b across patients who were between 18 and 65 years of age.

### Baseline measurements

#### Migraine characteristics

A total of 2886 patients were enrolled in the three placebo-controlled, double-blind phase 3 trials included in the efficacy analysis. There were 1344 patients (47%) who were aged ≤40 years, 849 patients (29%) between the ages of > 40 and ≤ 50, 336 patients (12%) between the ages of > 50 and ≤ 55, and 357 patients (12%) between the ages of > 55 and ≤ 65.

The baseline migraine characteristics of patients in the 4 age groups used to analyze efficacy outcomes are shown in Table 3. As expected, the number of years since diagnosis increased with increasing age for patients with episodic and chronic migraine. The numbers of monthly migraine headache days were similar for all age groups. Statistical comparison of the 4 age groups showed no significant difference between age subgroups for overall mean monthly migraine headache days in the episodic studies. However, in chronic migraine trials, the comparison of overall means between age groups showed some significant differences between the groups, though there did not appear to be a consistent trend of increasing or decreasing with age. There were also statistically significant differences among age groups for mean monthly migraine headache days with acute medication use, for both episodic and chronic migraine trials. While there was no discernable trend in patients with episodic migraine, patients with chronic migraine in age groups > 40 years old generally had higher means for monthly migraine headache days with acute medication use.

**Table 3:** Baseline migraine characteristics and patient function and disability for 4 age groups (efficacy groupings)

		Migraine Characteristics				Patient Function and Disability			
			Years since diagnosis	Monthly migraine headache days	Monthly migraine headache days with acute medication		MSQ-RFR score	PGI-S score	MIDAS total score
**Age group (years)**	**Treat.**	**N**	**Mean (SD)**	**Mean (SD)**	**Mean (SD)**	**N**	**Mean (SD)**	**Mean (SD)**	**Mean (SD)**
**Episodic**									
**≤40**	PBO 120 240	381 219 220	14.0 (7.6) 14.2 (8.5) 14.3 (7.7)	9.3 (2.9) 9.3 (3.0) 9.2 (2.9)	6.9 (3.5) 7.1 (3.6) 7.1 (3.4)	379 219 218	52.1 (15.9) 51.8 (15.4) 48.9 (17.1)^2^	4.2 (1.1)^1^ 4.2 (1.1) 4.3 (1.2)	33.1 (26.4) 29.9 (23.5) 37.9 (28.1)^2^
**> 40 to ≤ 50**	PBO 120 240	298 125 113	22.0 (11.5) 23.1 (10.4) 22.4 (11.8)	9.3 (3.1) 8.9 (2.7) 9.0 (2.9)	8.2 (3.3) 7.7 (3.1) 7.8 (3.0)	296 124 111	51.6 (14.4) 51.6 (15.7) 51.5 (15.7)	4.3 (1.2) 4.4 (1.1) 4.4 (1.2)	34.0 (31.4) 35.6 (31.0) 30.4 (24.6)
**> 50 to ≤ 55**	PBO 120 240	96 54 58	25.3 (12.8) 27.8 (12.2) 26.4 (13.1)	8.7 (3.0) 9.1 (2.9) 9.1 (3.2)	7.7 (3.5) 8.1 (3.3) 8.0 (3.3)	96 54 58	51.7 (17.1) 50.8 (16.3) 50.3 (17.3)	4.3 (1.3) 4.2 (1.4) 4.4 (1.2)	32.0 (23.1) 36.3 (38.7) 31.8 (33.7)
**> 55 to ≤ 65**	PBO 120 240	119 46 44	34.1 (14.0) 34.8 (13.9) 30.5 (14.6)	8.7 (3.0) 8.9 (3.4) 8.8 (3.0)	7.7 (3.2) 7.5 (3.9) 7.3 (3.3)	116 46 43	54.0 (16.3) 55.3 (13.8) 54.2 (15.2)	4.2 (1.2) 4.0 (1.1) 4.3 (1.1)	31.7 (36.6) 26.0 (23.3) 30.3 (29.2)
***p*-value**^3^			< 0.001	0.128	< 0.001		0.075	0.349	0.468
**Chronic**									
**≤40**	PBO 120 240	253 144 127	14.8 (8.2) 14.7 (8.8) 13.3 (8.2)	20.0 (4.6) 19.7 (4.2) 19.6 (4.6)	14.3 (7.1) 13.9 (6.5) 13.3 (6.2)	247 141 125	37.8 (16.7) 37.6 (17.0) 39.1 (17.1)	4.8 (1.3) 4.9 (1.2) 4.7 (1.2)	69.1 (53.6) 64.2 (51.7) 76.4 (66.6)
**> 40 to ≤ 50**	PBO 120 240	162 75 76	24.1 (11.3) 23.6 (11.9) 21.9 (12.1)	19.4 (4.5) 19.1 (4.3) 19.1 (4.6)	16.7 (5.7) 16.0 (5.8) 15.2 (6.3)	159 75 74	37.7 (18.3) 41.0 (18.1) 37.5 (18.3)	5.1 (1.2) 4.8 (1.3) 5.0 (1.5)	76.3 (67.7) 59.3 (45.9)^2^ 65.4 (60.8)
**> 50 to ≤ 55**	PBO 120 240	65 31 32	28.7 (13.5) 25.8 (14.5) 24.1 (11.9)	18.9 (3.9) 18.3 (4.7) 17.4 (4.3)	15.8 (5.6) 16.0 (6.7) 13.4 (5.6)	63 29 32	38.6 (16.7) 40.9 (17.8) 38.7 (13.9)	4.9 (1.2) 4.6 (1.2) 4.9 (1.2)	63.0 (49.5) 63.3 (51.1) 60.5 (48.9)
**> 55 to ≤ 65**	PBO 120 240	78 28 42	35.0 (12.9) 34.9 (13.6) 34.3 (11.7)	19.0 (5.2) 19.4 (4.4) 19.4 (4.6)	16.8 (6.5) 18.1 (4.0) 17.6 (5.7)	77 27 41	41.5 (16.7) 41.6 (16.4) 41.2 (18.9)	4.8 (1.3) 4.6 (1.5) 5.2 (1.4)	56.0 (49.7) 61.4 (47.5) 60.8 (71.8)
***p*-value**^3^			< 0.001	0.006	< 0.001		0.214	0.181	0.128

*Abbreviations*: *120* Galcanezumab 120 mg, *240* Galcanezumab 240 mg, *MIDAS* Migraine Disability Assessment, *MSQ-RFR* Migraine-specific quality-of-life questionnaire role function-restrictive domain, *PBO* Placebo, *PGI-S* Patient global impression of severity, *SD* Standard deviation, *Treat* Treatment

^1^Group included 380 patients

^2^*p*-value < 0.05 compared to PBO-treatment arm in same age group

^3^*p*-value compares overall means between age groups

#### Patient evaluations of migraine effects

The baseline scores for measures of function and disability, as well as disease severity, for patients in the 4 age groups used to analyze efficacy outcomes are shown in Table 3. Baseline scores were similar across age groups for MSQ role function restrictive domain, PGI-S, and MIDAS, in both episodic and chronic migraine trials (all interaction *p*-values > 0.05), though there were three instances where a galcanezumab treatment group had a baseline score that was statistically different from the mean score of the placebo group within an age group.

### Efficacy results

#### Changes in number of monthly migraine headache days and monthly migraine headache days with acute medication use

**Fig. 2:**
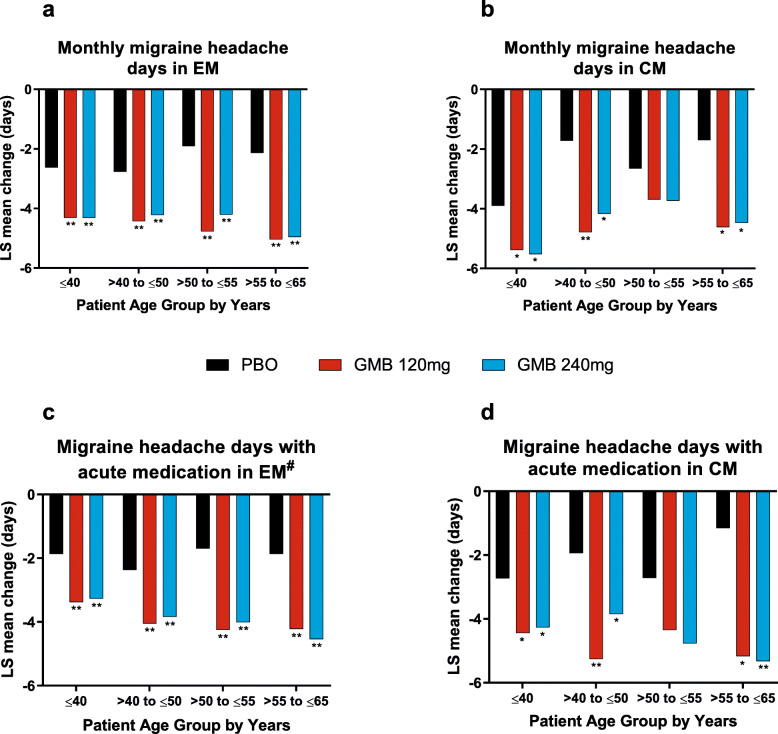
Reduction in migraine headache days and migraine headache days with acute medication, by age group

Overall mean changes from baseline in monthly migraine headache days and monthly migraine headache days with acute medication use in patients with episodic and chronic migraine are shown in Fig. 2. In patients with episodic migraine, galcanezumab treatment resulted in significantly greater reduction in monthly migraine headache days and monthly migraine headache days with acute medication use for all age groups (*p* < 0.001).

Age did not appear to affect changes in monthly migraine headache days for galcanezumab- versus placebo-treated patients, as treatment-by-subgroup interactions were not statistically significant for patients with either episodic or chronic migraine (interaction *p*-values > 0.05).

However, there was a significant treatment-by-subgroup interaction for change from baseline in monthly migraine headache days with acute medication use in patients with episodic migraine treated with galcanezumab 240 mg versus placebo, where it appeared that older patients treated with galcanezumab 240 mg had larger reductions than younger patients (interaction *p*-value = 0.024).

#### ≥50% and ≥75% improvement in monthly migraine headache days

**Fig. 3:**
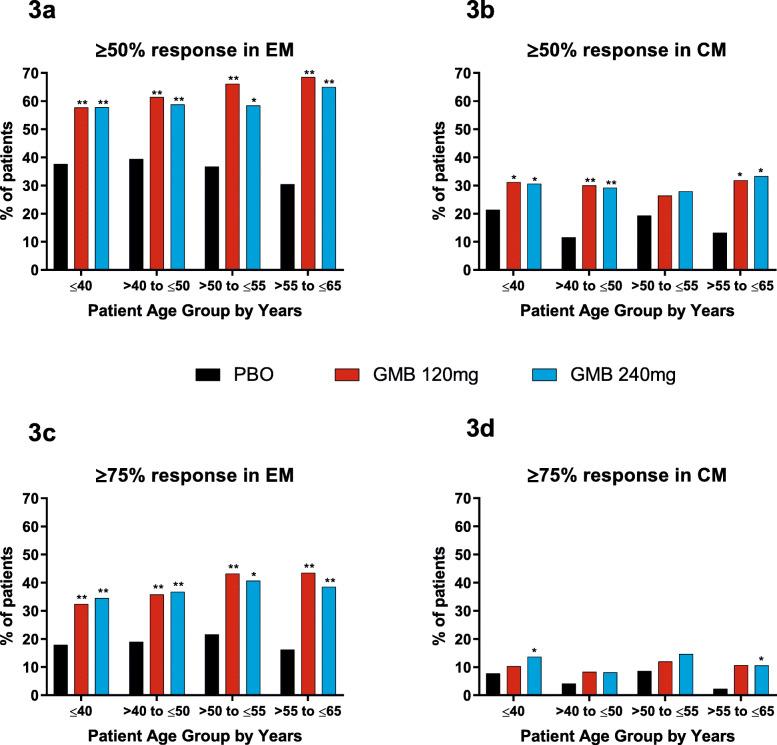
Percentages of patients with ≥50% and ≥75% reductions in migraine headache days, by age group

The percentages of patients experiencing ≥50% and ≥75% reductions from baseline in monthly migraine headache days are shown in Fig. 3. In patients with episodic migraine, people treated with galcanezumab experienced ≥50% and ≥75% reductions in monthly migraine headache days at significantly higher rates than people treated with placebo, for all age groups. Patients with chronic migraine also experienced ≥50% and ≥75% reductions at higher percentages when treated with galcanezumab compared with placebo, but the differences were statistically significant only for some age groups.

Age did not affect the likelihood of a patient achieving these reductions in monthly migraine headache days for galcanezumab- versus placebo-treated patients, as treatment-by-subgroup interactions were not significant in episodic and chronic studies (interaction *p*-values > 0.05, or not calculable for ≥75% reduction in patients with chronic migraine due to insufficient number of patients).

### Safety results

#### Safety population

In the Phase 3_Pooled group, there were 1429 patients (50%) who were aged < 42 years, 681 patients (24%) between the ages of 42 and < 50 years, 609 patients (21%) between the ages of 50 and < 60 years, and 167 patients (6%) between the ages of 60 and ≤ 65 years, who were evenly divided between galcanezumab and placebo treatment. Safety analyses were also conducted using the All GMB Exposure population, which included a total of 2586 galcanezumab-treated patients from the Phase 3_Pooled group, as well as from CGAB, Art-01, and CGAJ clinical trials, and also included data from the open-label phase of Study CGAI. In the All GMB Exposure group, there were 1295 patients (50%) who were aged < 42 years, 599 patients (23%) between the ages of 42 and < 50 years, 538 patients (21%) between the ages of 50 and < 60 years, and 154 patients (6%) between the ages of 60 and ≤ 65 years.

#### TEAEs, SAEs, and DCAEs

In the double-blind treatment phase, TEAEs were reported less frequently by patients ≥60 years old treated with galcanezumab versus placebo, as indicated by a lower incidence rate (EAIR) (Table 4). The incidence rate for galcanezumab-treated patients ≥60 years old was also numerically lower than rates seen in younger galcanezumab-treated age groups. No treatment-by-age subgroup interactions were observed for TEAEs (*p* = 0.097) or SAEs (*p* = 0.107) in the double-blind treatment phase population. There was a statistically significant treatment-by-age group interaction for DCAEs (*p* = 0.022), where placebo-treated patients ≥60 years had a higher incidence rate than all other age subgroups, but there was no numerical increase in DCAEs in galcanezumab-treated patients of the same age. Overall, patients aged ≥60 years who were treated with galcanezumab did not have increased incidences of TEAEs or DCAEs, and they were not more likely than patients in the 50 to < 60 age group to experience an SAE.

**Table 4:** Incidence rates for TEAEs, SAEs, and DCAEs

			Patients with One or More TEAE		Patients with One or More SAE		Patients with DCAE	
Dose Group	Age Group (years)	N	n/TPY	EAIR (95% CI)	n/TPY	EAIR (95% CI)	n/TPY	EAIR (95% CI)
**Double-blind treatment phase**								
**Phase 3_Pooled** **Placebo**	**< 42** **42 to < 50** **50 to < 60** **60 to ≤ 65**	687 359 309 96	397/134.7 197/78.1 177/64.3 56/18.1	294.8 (266.5, 325.2) 252.3 (218.3, 290.0) 275.2 (236.1, 318.8) 308.7 (233.2, 400.9)	8/248.1 0/136.2 2/112.8 4/33.3	3.2 (1.4, 6.4) 0.0 (NA, 2.7) 1.8 (0.2, 6.4) 12.0 (3.3, 30.7)	11/248.2 1/136.1 6/112.5 6/33.2	4.4 (2.2, 7.9) 0.7 (0.0, 4.1) 5.3 (2.0, 11.6) 18.1 (6.6, 39.3)
**Phase 3_Pooled** **GMB**	**< 42** **42 to < 50** **50 to < 60** **60 to ≤ 65**	742 322 300 71	472/143.2 202/64.9 198/54.9 41/15.8	329.6 (300.5, 360.7) 311.4 (269.9, 357.4)^1^ 360.5 (312.0, 414.3)^1^ 259.9 (186.5, 352.5)	10/272.1 2/122.7 9/110.8 2/25.9	3.7 (1.8, 6.8) 1.6 (0.2, 5.9) 8.1 (3.7, 15.4) 7.7 (0.9, 27.9)	22/272.3 7/122.2 5/112.2 1/26.4	8.1 (5.1, 12.2) 5.7 (2.3, 11.8) 4.5 (1.5, 10.4) 3.8 (0.1, 21.1)
**Treatment-by-age group interaction*p*-value**				0.097		0.107		0.022
**All GMB exposure**								
**All GMB Exposure**	**< 42** **42 to < 50** **50 to < 60** **60 to ≤ 65**	1295 599 538 154	913/308.3 409/165.2 381/130.3 110/42.7	296.2 (277.3, 316.0) 247.5 (224.1, 272.7) 292.5 (263.8, 323.4) 257.7 (211.8, 310.6)	31/709.4 12/358.1 18/311.4 5/89.0	4.4 (3.0, 6.2) 3.4 (1.7, 5.9) 5.8 (3.4, 9.1) 5.6 (1.8, 13.1)	51/714.3 18/359.9 18/313.8 4/91.0	7.1 (5.3, 9.4) 5.0 (3.0, 7.9) 5.7 (3.4, 9.1) 4.4 (1.2, 11.3)

*Abbreviations*: All GMB Exposure, patients treated with any GMB dose in any duration, *CI* Confidence interval, *DCAE* Discontinuation due to adverse event, *EAIR* Exposure-adjusted incidence rate, *GMB* Galcanezumab, *Phase 3_Pooled* all patients from the 3 placebo-controlled phase 3 trials, *SAE* Serious adverse event, *TEAE* Treatment-emergent adverse event, *TPY* Total patient years at risk

^1^*p* ≤ 0.05 compared with placebo

Incidence rates for the All GMB Exposure population were consistent across age groups and comparable to the values for the double-blind treatment phase population for TEAEs, SAEs, and DCAEs.

#### TEAEs likely to be cardiovascular in nature

The incidence rates for a selection of TEAEs likely to be cardiovascular in nature are shown in Table 5. The most commonly reported cardiovascular-related TEAE in all age groups was hypertension. In both the Phase 3_Pooled and All GMB Exposure population sets, the hypertension incidence rates for galcanezumab-treated patients in the 60 to ≤65 group were higher than the rates seen in the < 42 age group and 42 to < 50 age group. However, in the Phase 3_Pooled double-blind treatment phase population, which included a placebo-treated group, the hypertension incidence rate for galcanezumab-treated patients in the 60 to ≤65 age group was similar to that for the placebo-treated patients of the same age.

**Table 5:** Treatment-emergent adverse events likely to be cardiovascular in nature

			Hypertension (SMQ)^a^		Embolic and Thrombotic events (SMQ)^a^		Ischemic heart disease (SMQ)^a^	
Dose Group	Age Group (years)	N	n/TPY	EAIR (95% CI)	n/TPY	EAIR (95% CI)	n/TPY	EAIR (95% CI)
**Double-blind treatment phase**								
**Phase 3_Pooled** **Placebo**	**< 42** **42 to < 50** **50 to < 60** **60 to ≤ 65**	687 359 309 96	6/248.3 7/134.9 3/112.8 2/33.5	2.4 (0.9, 5.3) 5.2 (2.1, 10.7) 2.7 (0.6, 7.8) 6.0 (0.7, 21.6)	1/249.2 0/136.2 1/113.2 2/33.8	0.4 (0.0, 2.2) 0.0 (NA, 2.7) 0.9 (0.0, 4.9) 5.9 (0.7, 21.4)	0/249.3 0/136.2 0/113.3 1/33.9	0.0 (NA, 1.5) 0.0 (NA, 2.7) 0.0 (NA, 3.3) 3.0 (0.1, 16.4)
**Phase 3_Pooled** **GMB**	**< 42** **42 to < 50** **50 to < 60** **60 to ≤ 65**	742 322 300 71	5/273.7 2/122.5 7/110.8 2/26.0	1.8 (0.6, 4.3) 1.6 (0.2, 5.9) 6.3 (2.5, 13.0) 7.7 (0.9, 27.8)	1/274.0 1/122.8 2/112.3 0/26.6	0.4 (0.0, 2.0) 0.8 (0.0, 4.5) 1.8 (0.2, 6.4) 0.0 (NA, 13.9)	0/274.1 1/123.0 1/112.4 0/26.6	0.0 (NA, 1.4) 0.8 (0.0, 4.5) 0.9 (0.0, 5.0) 0.0 (NA, 13.9)
**All GMB exposure**								
**All GMB Exposure**	**< 42** **42 to < 50** **50 to < 60** **60 to ≤ 65**	1295 599 538 154	13/713.6 7/360.1 14/311.7 5/89.5	1.8 (1.0, 3.1) 1.9 (0.8, 4.0) 4.5 (2.5, 7.5) 5.6 (1.8, 13.0)	2/717.6 1/362.1 3/314.8 0/91.4	0.3 (0.0, 1.0) 0.3 (0.0, 1.5) 1.0 (0.2, 2.8) 0.0 (NA, 4.0)	0/718.4 1/361.6 2/314.8 0/91.4	0.0 (NA, 0.5) 0.3 (0.0, 1.5) 0.6 (0.1, 2.3) 0.0 (NA, 4.0)

*Abbreviations*: *All GMB Exposure* Patients treated with any GMB dose in any duration, *CI* Confidence interval, *EAIR* Exposure-adjusted incidence rate, *GMB* Galcanezumab, *NA* Not applicable, *Phase 3_Pooled* all patients from the 3 placebo-controlled phase 3 trials, *SMQ* Standardized Medical Dictionary for Regulatory Activities query, *TEAE* Treatment-emergent adverse event, *TPY* Total patient years at risk

^a^SMQ search included only narrow terms

Very few patients reported embolic and thrombotic events or ischemic heart disease, and no galcanezumab-treated patients ≥60 years old experienced either of these TEAEs. In both the Phase 3_Pooled and All GMB Exposure population sets, no patient ≥60 years treated with galcanezumab discontinued for a cardiovascular-related adverse event. Incidence rates for the All-GMB Exposure set were comparable to those of the double-blind treatment phase for hypertension, embolic and thrombotic events, and ischemic heart disease.

While there was some apparent increase in incidence rates for cardiovascular-related TEAEs with increasing age, there was no indication that galcanezumab treatment increased these rates. In the Phase 3_Pooled population, an analysis of all SMQs together showed no treatment-by-age group interaction (*p* = 0.449).

#### High blood pressure

The summary of incidence rates for high BP by age group is shown in Table 6. In the double-blind treatment phase set, incidence rates for high systolic BP increased with increasing age in both placebo- and galcanezumab-treated groups, up to the ≥50 to < 60 age group, at which point the effect appeared to plateau. Incidence rates for high diastolic BP increased noticeably from the < 42 age group to the 42 to < 50 age group and remained higher in all older age groups. However, for both high systolic and high diastolic BP, galcanezumab treatment caused no significant increase in incidence rate compared to the placebo treatment of the same age group for all age groups. Incidence rates in the All-GMB Exposure set were comparable to or lower than rates observed in the double-blind treatment phase set.

**Table 6:** High blood pressure by age group

			Systolic Blood Pressure High^a^		Diastolic Blood Pressure High^b^	
Dose Group	Age Group (years)	N	n/TPY	EAIR (95% CI)	n/TPY	EAIR (95% CI)
**Double-blind treatment phase**						
**Phase 3_Pooled Placebo**	< 42 42 to < 50 50 to < 60 60 to ≤65	656 354 305 95	10/244.4 11/134.2 16/109.9 5/33.3	4.1 (2.0, 7.5) 8.2 (4.1, 14.7) 14.6 (8.3, 23.7) 15.0 (4.9, 35.0)	31/240.6 33/131.4 28/109.0 7/32.2	12.9 (8.8, 18.3) 25.1 (17.3, 35.3) 25.7 (17.1, 37.1) 21.7 (8.7, 44.8)
**Phase 3_Pooled GMB**	< 42 42 to < 50 50 to < 60 60 to ≤65	728 321 295 70	16/269.5 8/121.6 20/109.1 4/25.6	5.9 (3.4, 9.6) 6.6 (2.8, 13.0) 18.3 (11.2, 28.3) 15.6 (4.3, 40.0)	43/265.4 31/117.4 25/108.5 9/25.3	16.2 (11.7, 21.8) 26.4 (17.9, 37.5) 23.0 (14.9, 34.0) 35.6 (16.3, 67.6)
**Treatment-by-age group interaction p-value**				0.802		0.734
**All GMB exposure**						
**All GMB Exposure**	< 42 42 to < 50 50 to < 60 60 to ≤65	1268 598 530 152	32/703.9 28/350.1 42/299.7 13/87.3	4.6 (3.1, 6.4) 8.0 (5.3, 11.6) 14.0 (10.1, 18.9) 14.9 (7.9, 25.5)	92/685.0 77/332.1 58/293.9 17/85.0	13.4 (10.8, 16.5) 23.2 (18.3, 29.0) 19.7 (15.0, 25.5) 20.0 (11.7, 32.0)

*Abbreviations*: *All GMB Exposure* Patients treated with any GMB dose in any duration, *CI* Confidence interval, *EAIR* Exposure-adjusted incidence rate, *GMB* Galcanezumab, *Phase 3_Pooled* All patients from the 3 placebo-controlled phase 3 trials, *TPY* Total patient years at risk

^a^High systolic blood pressure is defined as any postbaseline measurement ≥140 mmHg and a ≥20 mmHg increase from baseline

^b^High diastolic blood pressure is defined as any postbaseline measurement ≥90 mmHg and a ≥10 mmHg increase from baseline

Overall, there was no significant treatment-by-age group interaction for either systolic (*p* = 0.802) or diastolic (*p* = 0.734) high BP in the double-blind treatment phase set, indicating that higher incidence rates of high BP in the older age groups are an effect of age rather than treatment.

## Discussion

Several reports have shown that the clinical characteristics of migraine, such as attack severity and disease profile, can be different in older patients [10, 15, 16]. As a person with migraine ages, their brain can be metabolically and physically altered due to their disease [22–24]. Furthermore, age can influence patient behavior in a clinical trial. For example, a trial evaluating rizatriptan for the acute treatment of migraine found that older patients had lower responses to placebo [25].

Evaluation of baseline characteristics showed some differences between age groups. Interestingly, in patients with either episodic or chronic migraine, the ≤40 age group had, on average, fewer monthly migraine headache days with acute medication use than did patients in the three older groups, even though the ≤40 age group did not have fewer monthly migraine headache days on average. However, the youngest patient group had approximately seven monthly migraine headache days with acute medication, while the two middle age groups had means close to 8 and the oldest group had a mean of about 7.5 day; hence, the difference in acute medication use may not be clinically significant.

Age may affect rates of lymph flow and endocytosis, which can result in reduced absorption, distribution, or elimination of a mAb [26]. Pharmacokinetic studies with mAbs show mixed results regarding the effect of age, but limited data exist for young and elderly populations [27–31]. This analysis indicated that there was a minor reduction in CL/F with increasing age up to 65 years with no reduction in CL/F IIV. These findings suggest that dosing adult patients based on age to limit exposure variability would not be warranted for galcanezumab, and as such, per the product label, galcanezumab can be administered irrespective of age in an adult population [1, 2]. This study’s finding that age did not meaningfully influence blood concentrations of galcanezumab is consistent with no differential treatment effect in older age subgroups.

Elimination of a mAb from the body can occur by intracellular catabolism within the liver and kidney [32], so it is possible that mAb elimination may be altered in older patients since increasing age is associated with decreases in kidney and liver functions [12]. However, other organs including skin, muscle, and intestine are also involved in mAb degradation [32], thereby, possibly mitigating effects on mAb elimination due to renal or hepatic insufficiency. A previous study evaluated creatinine clearance, a measure of kidney function, and bilirubin concentration, a marker of liver function, across healthy adults and patients with migraine up to 65 years [20]. Results showed that the CL/F of galcanezumab was not affected by creatinine clearance (median: 111 mL/min (data on file); range: 24–308 mL/min) or bilirubin concentration (median: 7 μmol/L (data on file); range 2–46 μmol/L) [20]. Since most patients had normal to slightly above normal creatinine clearance and bilirubin levels, the effect of a more severe kidney or liver condition on galcanezumab PK in patients with migraine could not be sufficiently ascertained by this analysis.

Galcanezumab outperformed placebo on all efficacy measures, regardless of disease subtype and age group, although the treatment difference within age groups did not always reach statistical significance. In the chronic migraine > 50 to ≤55 age group, differences in efficacy between placebo and galcanezumab treatment were not statistically significant. This lack of significance was not likely due to advanced age because differences were significant for the older group (> 55 to ≤65 years) in chronic migraine but might have been due to a relatively high level of response to placebo treatment in this age group. In the efficacy analyses, the placebo response varied among age groups but without any trend that would suggest a clear relationship with age and was likely due to random variability.

In addition, differences between placebo and galcanezumab treatment in ≥75% response in chronic migraine were not significant for some age groups. Patients with chronic migraine had a mean baseline of approximately 19 monthly migraine headache days. Therefore, a decrease of ≥75% was a higher hurdle for this group compared to patients with episodic migraine, resulting in relatively smaller numbers of patients included in these calculations. Despite lack of statistical significance in some measures in patients with chronic migraine, there did not appear to be any trend suggesting that increasing age was associated with decrease in effect.

Results on the incidence rates for TEAEs, SAEs, and DCAEs showed a significant treatment-by-age group interaction only for DCAEs, but with no clear trend by age group to indicate that older age was associated with increased risk of discontinuing treatment due to an adverse event.

While incidence rates for hypertension, as well as for high systolic and diastolic BP, were higher in older patients, comparisons between placebo- and galcanezumab-treated patients showed that galcanezumab treatment did not increase the chance of these cardiac-related TEAEs.

The aim of this study was to investigate the PK, efficacy, and safety of galcanezumab in older patients. However, patients over the age of 55 years made up a relatively small fraction of the total patients enrolled in the included studies. Of the 2886 patients in the placebo-controlled phase 3 trials, 357 (12.4%) were between the ages of 55 and 65 years and 167 (5.8%) patients were over 60 years of age. The inclusion of three additional trials in the safety data contributed another 83 galcanezumab-treated patients between 60 and 65 years to these analyses. Despite making up a smaller portion of the trial patients, we believe that the numbers of patients in the older age groups were sufficient to demonstrate maintained efficacy of galcanezumab in older patients, as well as support a level of safety equal to that seen in younger patients.

While migraine can be experienced by people of all ages [7, 10], the studies analyzed here enrolled patients with a maximum age of 65 years. However, the safety of galcanezumab in an older population was also addressed in a previous study of galcanezumab in patients with osteoarthritis. This earlier study enrolled patients between 40 and 75 years of age, with a mean age of 59 years, which is almost 18 years older than the mean age for patients enrolled in the placebo-controlled episodic and chronic migraine trials. Despite the inclusion of older patients in the osteoarthritis study, galcanezumab treatment was not associated with any changes in diastolic or systolic BP or clinically significant changes in safety laboratory tests [33].

Another limitation of this study is that patients with acute cardiovascular events within 6 months of screening, including those who had experienced stroke, myocardial infarction, unstable angina, percutaneous coronary intervention, coronary artery bypass graft, deep vein thrombosis/pulmonary embolism, or had planned cardiovascular surgery or percutaneous coronary angioplasty, or other serious cardiovascular risks were not included in these galcanezumab clinical trials. Older people, including those with migraine, especially those over the age of 65 years, have a higher risk of serious cardiovascular events [34], and this study was not able to consider patients with these medical conditions.

Age was not a stratification factor in any of the placebo-controlled phase 3 trials, so there are some imbalances of age subgroups across the treatment arms in these trials in episodic and chronic migraine. However, imbalances are expected to be mitigated by randomization, and the analyses by age appropriately account for the age main effect in the model to avoid any potential confounding. The relatively small sample size of some age subgroups potentially limited the power of the analyses, and this limitation may account for the lack of significance seen in some comparisons, particularly in the analyses of chronic migraine outcomes.

## Conclusions

Overall, there was no indication that patient age (up to 65 years) affects efficacy of galcanezumab in migraine prevention, nor does it influence galcanezumab PK to an extent that would necessitate age-related dose adjustment. Furthermore, older patients did not experience increases in frequency of adverse events or increases in BP when treated with galcanezumab as compared with placebo.

## Data Availability

Lilly provides access to all individual participant data collected during the trial, after anonymization, with the exception of pharmacokinetic or genetic data. Data are available to request 6 months after the indication studied has been approved in the US and EU and after primary publication acceptance, whichever is later. No expiration date of data requests is currently set once data are made available. Access is provided after a proposal has been approved by an independent review committee identified for this purpose and after receipt of a signed data sharing agreement. Data and documents, including the study protocol, statistical analysis plan, clinical study report, blank or annotated case report forms, will be provided in a secure data sharing environment. For details on submitting a request, see the instructions provided at **www.vivli.org****.**

## Abbreviations

BP: Blood pressure
CGRP: Calcitonin gene-related peptide
CL/F: Apparent clearance
DCAE: Discontinuation due to adverse event
EAIR: Exposure-adjusted incidence rate
IIV: Inter-individual variability
mAb: Monoclonal antibody
MIDAS: Migraine Disability Assessment
MOF: Model of the objective function
MSQ: Migraine-Specific Quality-of-life Questionnaire v2.1
PGI-S: Patient global impression of severity
PK: Pharmacokinetics
SAE: Serious adverse event
SMQ: Standardized Medical Dictionary for Regulatory Activities query
TEAE: Treatment-emergent adverse event
V/F: Apparent volume of distribution.
